# Treatment of Pneumonia

**Published:** 1869-04

**Authors:** John Addington Symonds


Treatment of Pneumonia.—By John Addington Symonds, M. D., F. R. C. S. E. in *British Medical Journal*, June 13.

Full and frequently repeated doses of bicarbonate of potass and spirit of nitric ether, with antimonial wine or ipecacuanah, in combination with poultices or blisters, are Dr Symond's first remedies. A serious illustration of an untimely opiate suddenly causing a fatal relapse, or rather a fatal arrest of the resolution, occurred to Dr. Symonds a few years ago, in the case of a gentleman whose pleuro-pneumonia was subsiding with free expectoration of muco-purulent matter. From coughing, or some other exertion, which probably caused a partial extension of pleurisy, he suffered an attack of pain, for which he helped himself to so heavy a dose of laudanum as to induce serious narcotism. The narcotism passed off, but the expectoration could not be recalled; and the patient died of his intolerance of pain, or, rather, of his rashness in quelling it.

